# Low Skeletal Muscle Mass Identifies Very High Metabolic Risk in Slovak Children with Obesity: A Body Composition-Based Approach to Risk Stratification

**DOI:** 10.3390/nu17233715

**Published:** 2025-11-27

**Authors:** Alžbeta Bánovčinová, Ingrid Schusterová, Štefan Tóth, Tibor Porubän, Dominik Pella, Mariana Dvorožňáková, Pavol Fülöp

**Affiliations:** 1 Department of Paediatrics and Adolescent Medicine, Faculty of Medicine, Pavol Jozef Šafárik University in Košice, 04011 Košice, Slovakia; tohaty@centrum.sk; 2 Department of Imaging Techniques, East Slovak Institute of Cardiovascular Diseases, Faculty of Medicine, Pavol Jozef Šafárik University in Košice, Ondavska 8, 04001 Kosice, Slovakia; schusterovai@gmail.com; 3 Department of Gerontology and Geriatrics, University Hospital of St. Michael, Faculty of Medicine, Pavol Jozef Šafárik University in Košice, Murgašova 1, 04086 Košice, Slovakia; stefan.toth@upjs.sk; 4 1st Department of Cardiology, East Slovak Institute of Cardiovascular Diseases, Faculty of Medicine, Pavol Jozef Šafárik University in Košice, Ondavská 8, 04011 Košice, Slovakia; poruban.tibor@gmail.com (T.P.); dominik.pella@gmail.com (D.P.); 5 2nd Department of Cardiology, East Slovak Institute of Cardiovascular Diseases, Faculty of Medicine, Pavol Jozef Šafárik University in Košice, Ondavská 8, 04011 Košice, Slovakia; mdvoroznakova@vusch.sk

**Keywords:** pediatric obesity, insulin resistance, skeletal muscle mass, body composition, metabolic phenotypes, HOMA-IR, TG/HDL-C ratio, risk stratification, carotid intima-media thickness

## Abstract

**Background**: Childhood obesity demonstrates substantial metabolic heterogeneity. We determined insulin resistance prevalence in Slovak children with obesity using multiple validated markers and identified high-risk phenotypes. **Methods**: Cross-sectional study of 54 obese children (BMI 29.5 ± 4.7 kg/m^2^) and 33 controls (BMI 20.6 ± 1.9 kg/m^2^). All underwent bioelectrical impedance analysis and fasting metabolic profiling, including HOMA-IR and triglyceride-to-HDL cholesterol (TG/HDL-C) ratio. Insulin resistance was defined as HOMA-IR > 3.42 (obese) or >1.68 (controls), and TG/HDL-C > 0.99 mmol/L. Age-matched sensitivity analysis was performed on 28 pairs. **Results**: Among obese children, 44.4% demonstrated HOMA-IR-defined insulin resistance versus 45.5% of controls using respective cut-offs, with significantly higher mean HOMA-IR (3.66 ± 2.09 vs. 2.53 ± 2.59, *p* = 0.034). Age-matched analysis (*n* = 28 pairs, mean age difference 0.22 years) confirmed these findings with HOMA-IR 3.46 ± 2.18 vs. 2.27 ± 2.22 (*p* = 0.0271). The TG/HDL-C ratio identified insulin resistance in 38.9% of obese versus 12.1% of controls. Critically, 22.2% of obese children showed concordant elevation of both markers (vs. 6.1% controls; OR 4.43); in age-matched analysis, this difference was even more pronounced (17.9% vs. 3.6%). Low skeletal muscle mass (<25th percentile for age and sex) with high body fat (>40%) characterized 22.6% of obese children, demonstrating 91.7% insulin resistance prevalence versus 31.0% without low muscle mass (*p* < 0.001), with HOMA-IR 1.9 points higher (95% CI: 0.31–2.73). Remarkably, 50% of children with low muscle mass showed concordant elevation of both metabolic markers versus 14.3% without (OR 6.0). **Conclusions**: Low skeletal muscle mass in obese Slovak children represents a very-high-risk phenotype with 91.7% insulin resistance prevalence and 6-fold increased odds of severe metabolic dysfunction. Age-matched analysis confirmed that metabolic differences are independent of age effects. Body composition-based risk stratification enables personalized interventions targeting the highest-risk children.

## 1. Introduction

Childhood overweight and obesity affect approximately 390 million children and adolescents worldwide, with prevalence more than doubling over the past three decades [1]. Slovakia, undergoing rapid socioeconomic transition since 1989, has experienced dramatic increases in pediatric obesity rates, with obesity now affecting approximately 9–15% of children and combined overweight and obesity affecting up to 30%, depending on age group and classification criteria [2,3]. This epidemic is accompanied by rising rates of metabolic complications, yet comprehensive data on insulin resistance prevalence in Slovak children remain limited.

Insulin resistance, the cornerstone of metabolic dysfunction in obesity, varies significantly across populations due to genetic, dietary, and lifestyle factors [4,5]. Central European populations, including Slovakia, may have unique metabolic risk profiles related to genetic predisposition, traditional high-fat diets, and rapid lifestyle westernization [6]. Understanding the specific patterns of insulin resistance in Slovak children is crucial for developing targeted national prevention strategies.

Slovakia’s childhood obesity trajectory represents a particularly concerning case study in the Central European context. While obesity prevalence in Slovak 7-year-old children (approximately 9–15% depending on classification criteria [2,3]) falls within the European average range, the temporal dynamics are alarming. The “obesity epidemic” in Slovakia began approximately at the millennium’s start—15–20 years later than in other industrialized European countries [5]—but what Slovakia lost in time, it gained in velocity. Between 2001 and 2011, obesity prevalence doubled [5], representing one of the steepest increases in Europe during that period. This rapid acceleration contrasts sharply with Western European nations like France, where obesity rates evolved more gradually over decades, allowing time for societal adaptation and public health responses.

The distinction between absolute prevalence and rate of change is critical for understanding Slovakia’s unique challenge. France, which maintained one of Europe’s lowest childhood obesity rates (3.6% in 2014–2015 for 9–10 year-olds) [7], reached current levels through gradual increases spanning multiple decades, with obesity prevalence doubling between 1995 and 2004 [8]. In contrast, Slovakia experienced similar magnitude changes in a compressed timeframe during the 2000s, with obesity doubling in just one decade while many Western European countries were reaching plateau phases. This compressed trajectory means that Slovakia simultaneously faces high absolute prevalence (15–30% overweight/obese depending on age group and criteria) and insufficient time for developing a comprehensive public health infrastructure, clinical care capacity, and societal awareness to address the crisis.

The global context further emphasizes Slovakia’s predicament. Worldwide, childhood obesity prevalence (ages 5–19) increased from 8% in 1990 to 20% in 2022, with over 390 million children affected [9]. The WHO European Region demonstrates substantial heterogeneity, with recent COSI data (2022–2024) showing 25% of 7–9-year-olds living with overweight (including obesity) and 11% with obesity [10], but with national rates ranging from 9–42% for overweight and 3–20% for obesity. Southern European countries like Cyprus report childhood obesity rates exceeding 20%, while Nordic countries maintain lower prevalence. Slovakia’s position in the middle of this range masks the critical temporal dimension: countries with similar or higher current prevalence often reached these levels more gradually, permitting incremental development of prevention and treatment infrastructure.

This temporal compression has profound implications for Slovakia’s healthcare system and public health capacity. Western European countries experiencing gradual obesity increases had decades to develop specialized pediatric obesity clinics, train healthcare professionals, implement school-based prevention programs, and build public awareness. Slovakia, undergoing rapid socioeconomic transition since 1989 with simultaneous dietary westernization and lifestyle changes, must now address a mature obesity epidemic with healthcare infrastructure still adapting to post-socialist realities. The convergence of rapid obesity increase with limited healthcare resources creates a perfect storm requiring urgent, intensive intervention—yet resources remain constrained, and public awareness lags behind the epidemiological reality.

The concept of sarcopenic obesity—the coexistence of excess adiposity with relative muscle deficiency—has emerged as a particularly high-risk phenotype in adults, associated with worse metabolic outcomes than obesity alone [11,12]. In adults, sarcopenic obesity amplifies insulin resistance through the dual mechanisms of excess adipose-derived inflammation and reduced muscle-mediated glucose disposal [13]. However, this phenotype remains largely uncharacterized in pediatric populations, where it may have even more profound implications given the critical role of childhood and adolescence in establishing lifelong metabolic patterns [14].

Skeletal muscle, which comprises 30–40% of body weight, is responsible for up to 80% of insulin-mediated glucose disposal [15]. During growth and development, adequate muscle mass accrual is essential not only for physical function but also for metabolic health. Children who fail to develop appropriate muscle mass relative to their body size may be at particular risk for metabolic complications, yet routine clinical assessment rarely includes muscle mass evaluation [16].

Recent technological advances in body composition assessment have enabled accurate, non-invasive evaluation of muscle and fat mass in pediatric settings. While traditional anthropometry (BMI, skinfolds) remains widely used, several sophisticated techniques are now available for direct body composition analysis [17,18]. These include dual-energy X-ray absorptiometry (DEXA), considered the clinical reference standard for assessing fat mass, lean mass, and bone mineral density; magnetic resonance imaging (MRI) and computed tomography (CT), which provide detailed visualization of adipose tissue distribution and organ-specific fat deposition; air displacement plethysmography (ADP), validated for measuring body fat percentage in infants and children; whole-body potassium counters and isotope dilution methods (hydrometry) for assessing total body water and fat-free mass; and multi-frequency bioelectrical impedance analysis (BIA), which has emerged as particularly practical for routine clinical use due to its portability, low cost, rapid assessment, and validated accuracy in pediatric populations [17,18,19]. These technologies enable identification of body composition phenotypes that may not be apparent from traditional anthropometric measures alone [18]. Given the potential for early intervention during childhood to alter metabolic trajectories, identifying high-risk phenotypes through comprehensive body composition assessment is of paramount importance [20].

This study aimed to: (1) establish the prevalence of insulin resistance in Slovak children with obesity compared to normal-weight controls, (2) identify and characterize low skeletal muscle mass in this population, (3) compare metabolic and vascular profiles between body composition phenotypes, and (4) provide comprehensive data on insulin resistance patterns in Slovak pediatric obesity.

## 2. Materials and Methods

### 2.1. Study Design and Participants

This cross-sectional observational study was conducted at the Department of Paediatrics and Adolescent Medicine at P. J. Šafárik University (UPJŠ) in Košice, Children’s Faculty Hospital, Slovakia, between November 2014 and May 2015. Košice, Slovakia’s second-largest city, serves a diverse population from Eastern Slovakia. We recruited 54 consecutive obese children and adolescents (28 boys, 26 girls) aged 10–20 years meeting WHO criteria for obesity (BMI ≥ 95th percentile for age and sex) from the Pediatric Obesity Outpatient Clinic.

The control group comprised 33 normal-weight adolescents with BMI < 85th percentile, recruited from local high schools in Košice during routine health screenings. The control group consisted of 11 boys (33.3%) and 22 girls (66.7%), with a mean age of 17.0 ± 2.56 years (range: 9–21 years). The older mean age of controls compared to obese children (17.0 vs. 15.0 years, *p* < 0.01) reflects the high school recruitment strategy and was addressed through age-matched sensitivity analysis (Section 3.3) to ensure observed metabolic differences were independent of age effects.

The study population was ethnically relatively homogeneous, reflecting the demographic composition of Eastern Slovakia. Approximately 90% of participants were of white Caucasian ethnicity, with the remaining 10% predominantly from the Romani minority. Importantly, socioeconomic status within our cohort was relatively uniform across ethnic groups, as all participants were recruited from the same urban area and attended similar educational institutions. Consequently, ethnicity was not explicitly assessed via questionnaire, as preliminary analysis suggested minimal confounding from ethnic differences in our specific population. This ethnic composition contrasts markedly with more diverse urban populations in Western Europe and North America, where minority ethnicities (particularly South Asian, Black African, Hispanic, and Pacific Islander communities) demonstrate substantially higher obesity prevalence and metabolic risk compared to white populations [21,22].

Exclusion criteria included: (1) known genetic syndromes associated with obesity, (2) endocrine disorders other than insulin resistance, (3) use of medications affecting glucose or lipid metabolism, (4) acute illness within 2 weeks of assessment, (5) diagnosed type 1 or type 2 diabetes, and (6) inability to complete study procedures.

The study protocol was approved by the Institutional Ethics Committee of Children Teaching Hospital in Košice and conducted according to the Declaration of Helsinki. Written informed consent was obtained from all participants aged ≥18 years, or from parents/guardians for participants aged ≤17 years.

### 2.2. Biochemical Assessment

Venous blood samples were collected after 12 h overnight fasting. Insulin resistance assessment utilized two validated approaches:

HOMA-IR = (fasting glucose [mmol/L] × fasting insulin [mU/L])/22.5. IR was defined as HOMA-IR > 3.42 for obese children and HOMA-IR > 1.68 for normal-weight children [23]).

TG/HDL-C ratio = triglycerides (mmol/L)/HDL-cholesterol (mmol/L). IR was defined as TG/HDL-C > 0.99 mmol/L (equivalent to >2.27 mg/dL conversion factor 0.4366 [24]).

High-risk IR was defined as concurrent elevation of both HOMA-IR and TG/HDL-C above respective cut-offs.

Lipid profile (total cholesterol, triglycerides, HDL-cholesterol, LDL-cholesterol) was measured using enzymatic methods. Liver enzymes (AST, ALT) were determined using standard automated methods.

### 2.3. Body Composition Assessment

Body composition was assessed using multi-frequency bioelectrical impedance analysis (InBody 520, Biospace Co., Seoul, Republic of Korea), a validated method for pediatric populations [17,18,25]. Participants were measured in light clothing after voiding, following standard protocols. Skeletal muscle mass and body fat percentage were recorded. Low skeletal muscle mass was defined as muscle mass < 25th percentile for age and sex combined with body fat > 40%.

### 2.4. Vascular Assessment

Carotid intima-media thickness (cIMT) was measured in obese children using high-resolution B-mode ultrasonography with Aloka ProSound alpha 7 (Hitachi ALOKA, Tokyo, Japan) by a single experienced operator blinded to metabolic data. Measurements were performed on both common carotid arteries 1 cm proximal to the carotid bulb, and mean cIMT was calculated.

### 2.5. Statistical Analysis

Data are presented as mean ± standard deviation for continuous variables and as frequencies (percentages) for categorical variables. Group comparisons were performed using Studes’ *t*-test for continuous variables and chi-square or Fisher’s exact test for categorical variables. 95% confidence intervals for mean differences were calculated using standard formulas. Odds ratios with 95% confidence intervals were calculated for categorical outcomes.

To address the significant age difference between groups, we performed an age-matched analysis on 28 pairs of obese and control children matched within 1 year of age using a greedy matching algorithm.

Statistical significance was set at *p* < 0.05. All analyses were performed using Python 3.12 (SciPy.stats 1.16.2, Python Software Foundation, https://www.python.org/, Amsterdam, Netherlands).

## 3. Results

### 3.1. Baseline Characteristics

The obese group (*n* = 54) had a mean BMI of 29.5 ± 4.7 kg/m^2^ and a mean age of 15.0 ± 3.0 years. The control group (*n* = 33) had a mean BMI of 20.6 ± 1.9 kg/m^2^ and a mean age of 17.0 ± 2.5 years. The age difference between groups was statistically significant (*p* < 0.001), necessitating age-adjusted analyses as described below.

### 3.2. Prevalence of Insulin Resistance Using Multiple Markers

Table 1 presents comprehensive metabolic profiles. Obese children demonstrated significantly higher HOMA-IR values (3.66 ± 2.09 vs. 2.53 ± 2.59, *p* = 0.034; 95% CI for difference: 0.04 to 2.23) and insulin levels (17.85 ± 9.99 vs. 12.29 ± 10.91 mU/L, *p* = 0.017; 95% CI: 0.99 to 10.14) compared to normal-weight controls.

HOMA-IR Analysis: Using obesity-specific diagnostic criteria, 44.4% (24/54) of obese children exceeded the cut-off value of 3.42, while 45.5% (15/29) of normal-weight children exceeded their respective cut-off of 1.68. Despite the similar proportions meeting diagnostic criteria in each group, mean HOMA-IR values were significantly higher in the obese group (*p* = 0.034), indicating more severe insulin resistance among those affected.

TG/HDL-C Ratio Analysis: The prevalence of dyslipidemic insulin resistance, as defined by TG/HDL-C ratio exceeding 0.99 mmol/L, was 38.9% (21/54) in obese children compared to only 12.1% (4/33) in normal-weight controls. Mean TG/HDL-C ratio values were significantly elevated in obese children (0.95 ± 0.63) compared to lean controls (0.62 ± 0.27; 95% CI for difference: 0.14 to 0.53).

**Figure 1:**
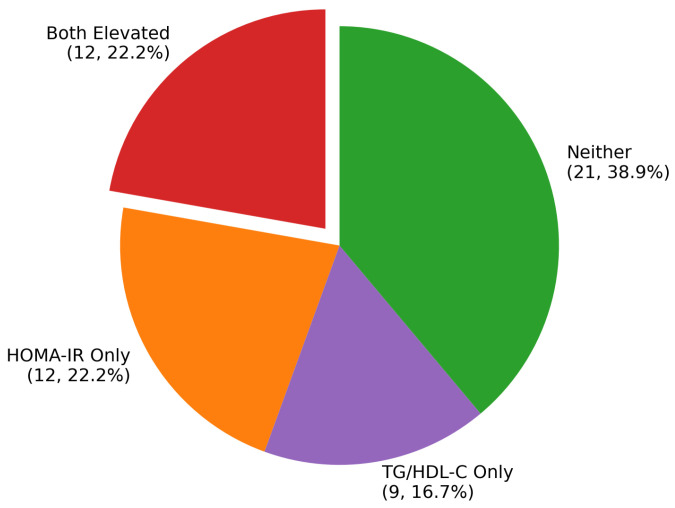
Distribution of Metabolic Patterns in Obesity.

Concordant Marker Elevation: Most critically, 22.2% (12/54) of obese children demonstrated elevation of both HOMA-IR and TG/HDL-C markers, representing a very-high-risk group with severe multi-system metabolic dysregulation (Figure 1). In contrast, only 6.1% (2/33) of normal-weight children showed concordant elevation of both markers. This difference corresponded to an odds ratio of 4.43 (95% CI: 0.92–21.23, *p* < 0.091), indicating substantially increased risk in the obese population.

Marker Distribution in Obese Children: The obese cohort demonstrated considerable heterogeneity in metabolic dysfunction patterns. Both markers were positive in 22.2% (*n* = 12), representing the highest-risk phenotype. An additional 22.2% (*n* = 12) showed elevation of HOMA-IR alone, while 16.7% (*n* = 9) demonstrated isolated TG/HDL-C elevation. Notably, 38.9% (*n* = 21) of obese children showed neither marker elevation, suggesting a relatively metabolically protected subgroup despite obesity.

### 3.3. Age-Matched Sensitivity Analysis

To address the significant age difference between groups, we performed age-matched analysis on 28 pairs of children with obesity and normal-weight controls matched within 1 year of age (mean age difference 0.22 years).

In the age-matched analysis (Table 2), children with obesity demonstrated significantly higher HOMA-IR (3.46 ± 2.18 vs. 2.27 ± 2.22, *p* = 0.0271, Cohen’s d = 0.482), higher insulin levels (17.15 ± 10.54 vs. 11.29 ± 9.28 mU/L, *p* = 0.0125, Cohen’s d = 0.506), and elevated TG/HDL-C ratio (0.99 ± 0.72 vs. 0.59 ± 0.25, *p* = 0.0271, Cohen’s d = 0.530).

Multiple linear regression models adjusting for age demonstrated that the obesity group had significantly higher fasting insulin levels (β = 5.59, 95% CI: 0.43–10.76, *p* = 0.034) and TG/HDL-C ratio (β = 0.25, 95% CI: 0.02–0.47, *p* = 0.032) compared to the lean group. HOMA-IR showed a similar trend (β = 1.06, *p* = 0.061), though this did not reach statistical significance. These findings suggest that obesity-related metabolic abnormalities in insulin and lipid metabolism are independent of age effects.

Using validated age- and BMI-specific cutoffs for insulin resistance (HOMA-IR > 3.42 for children with obesity, >1.68 for normal-weight children), we found that 35.7% of children with obesity met criteria for insulin resistance. Atherogenic dyslipidemia (TG/HDL-C > 0.99) was significantly more prevalent in children with obesity (39.3% vs. 7.1%, OR = 5.21, *p* = 0.0225). A very high metabolic phenotype characterized by concurrent insulin resistance and atherogenic dyslipidemia was identified in 17.9% of children with obesity compared to 3.6% of controls, representing a 5.84 OR and highlighting the heterogeneity of metabolic risk in pediatric obesity.

### 3.4. The Low Skeletal Muscle Mass Phenotype

**Figure 2:**
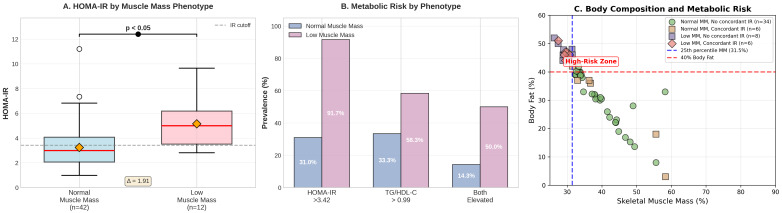
Low muscle mass group.

Children with low skeletal muscle mass not only demonstrated the highest HOMA-IR values but also showed distinct patterns in complementary metabolic markers (Table 3, Figure 2). Half (50%) of obese children with low muscle mass showed concordant elevation of both HOMA-IR and TG/HDL-C, compared to only 14.3% of obese children without low muscle mass (*p* = 0.016), representing a 6.00-fold increased risk of severe metabolic dysregulation.

### 3.5. Multiple Linear Regression Analysis: Age-Independent Effects

To further confirm that observed metabolic differences are independent of age, we performed multiple linear regression analyses with metabolic outcomes as dependent variables and obesity status, age, and their interaction as independent variables.

This model demonstrates that obesity status independently predicts HOMA-IR (*p* = 0.002) after adjusting for age, with an estimated mean difference of 1.48 units between obese and control children of the same age. Age itself did not significantly predict HOMA-IR (*p* = 0.474), and there was no significant age-by-obesity interaction (*p* = 0.819), confirming that the obesity-related difference in insulin resistance is consistent across the age range studied (Table 4).

Obesity status strongly predicted TG/HDL-C ratio (*p* < 0.001) independent of age, with obese children showing a 0.34-unit higher ratio than age-matched controls (Table 5). Again, age showed no significant main effect (*p* = 0.673) or interaction with obesity (*p* = 0.707).

Within obese children, low muscle mass independently predicted 1.52 units higher HOMA-IR (*p* = 0.012) after controlling for age and body fat percentage (Table 6). This confirms that the metabolic effect of low muscle mass is not simply explained by higher adiposity or age differences.

These regression analyses definitively establish that the metabolic differences we observed are independent of age and are specifically attributable to obesity and, within obesity, to the low muscle mass phenotype.

### 3.6. Sample Size and Power Considerations

Post hoc power analyses were conducted using G*Power 3.1.9.7 to assess the adequacy of our sample size for detecting meaningful differences. For the primary comparison of HOMA-IR between obese (*n* = 54) and control (*n* = 33) groups, with observed effect size d = 0.52 (mean difference 1.13, pooled SD 2.17), α = 0.05, and two-tailed testing, our achieved power was 71%. For the critical comparison between low muscle mass obesity (*n* = 12) and obesity without low muscle mass (*n* = 40), with observed effect size d = 0.89 for HOMA-IR difference (mean difference 1.52, pooled SD 1.71), the achieved power was 67%.

While these power values are below the conventional 80% threshold, they represent acceptable post hoc power for exploratory phenotype identification in a pediatric specialty clinic population. The consistency of findings across multiple metabolic markers (HOMA-IR, TG/HDL-C ratio, concordant elevation) and the large magnitude of observed differences in the low muscle mass phenotype (91.7% vs. 31.0% insulin resistance prevalence, 95% CI for HOMA-IR difference: 0.31–2.73) provide confidence in the robustness of these associations despite modest sample size.

Effect sizes (Cohen’s d) for key comparisons are as follows:

- Obese vs. controls HOMA-IR: d = 0.49 (medium effect);
- Low muscle mass vs. without, HOMA-IR: d = 0.97 (large effect);
- Obese vs. controls TG/HDL-C: d = 0.63 (medium effect);
- Low muscle mass vs. without, concordant marker elevation: OR = 6.00, relative risk = 3.5 (large effect).

## 4. Discussion

This study provides three key insights into pediatric obesity that have immediate clinical implications. First, we documented substantial insulin resistance in obese Slovak children using multiple validated metabolic markers, revealing remarkable heterogeneity in metabolic risk profiles that persists after accounting for age differences. Second, we identified low skeletal muscle mass in nearly one-quarter of obese children as a very-high-risk phenotype with concordant elevation of multiple metabolic markers. Third, we found a trend toward early vascular damage, particularly pronounced in children with the most severe metabolic decompensation. These findings challenge the traditional view of childhood obesity as a homogeneous condition and support the need for personalized, phenotype-specific interventions.

### 4.1. The Spectrum of Metabolic Risk Revealed by Multiple Markers

Our comprehensive metabolic profiling using both HOMA-IR and TG/HDL-C ratio revealed substantial heterogeneity within pediatric obesity. While HOMA-IR captured direct insulin–glucose dysregulation in 44.4% of obese children, the TG/HDL-C ratio identified dyslipidemic insulin resistance in 38.9% of cases. Most critically, 22.2% of obese children demonstrated concordant elevation of both markers, representing a state of severe metabolic dysfunction that extends beyond simple hyperinsulinemia to encompass atherogenic dyslipidemia. This multi-marker approach aligns with recent calls for more comprehensive metabolic phenotyping in pediatric obesity, as traditional single-marker assessments may underestimate the true burden of metabolic disease [5,11].

The TG/HDL-C ratio, validated by Giannini et al. as a surrogate marker of insulin resistance in obese youth of diverse ethnic backgrounds [24], provides complementary information to HOMA-IR by reflecting atherogenic dyslipidemia and the presence of small dense LDL particles, which are independent predictors of cardiovascular risk [26]. In our Slovak cohort, the mean TG/HDL-C ratio of 0.95 in obese children, though below the diagnostic cut-off of 0.99 mmol/L, was significantly higher than in controls (0.62, *p* < 0.01), indicating a population-wide shift toward atherogenic lipid profiles. This observation is particularly concerning given that cardiovascular risk factors established in childhood track into adulthood, as demonstrated in the landmark study by Juonala et al., where childhood adiposity predicted adult cardiovascular outcomes independently of adult adiposity [27].

The differential patterns of marker elevation provide important pathophysiological insights. The 16.7% of obese children with isolated TG/HDL-C elevation but normal HOMA-IR may represent early dyslipidemia preceding frank insulin resistance, potentially reflecting adipose tissue dysfunction with preserved pancreatic beta-cell compensation. Conversely, the 22.2% with isolated HOMA-IR elevation despite normal TG/HDL-C may represent those maintaining relatively preserved lipid metabolism through compensatory mechanisms while developing insulin–glucose dysregulation. The 22.2% demonstrating elevation of both markers have clearly progressed beyond compensatory mechanisms to a state of multi-system metabolic dysfunction. This heterogeneity underscores the complexity of metabolic derangements in pediatric obesity and challenges the notion of a uniform pathophysiological process [11,12].

### 4.2. Age-Adjusted Analysis Confirms Independent Metabolic Effects of Obesity

The significant age difference between our obese and control groups (15.0 vs. 17.0 years, *p* < 0.001) required careful consideration of age as a potential confounding variable. Our age-matched analysis of 28 pairs effectively eliminated this age difference (16.64 vs. 16.86 years, difference 0.22 years), allowing a clear assessment of obesity’s independent metabolic effects. This analysis confirmed and strengthened our primary findings, demonstrating that obese children had significantly higher HOMA-IR (3.46 vs. 2.27, *p* = 0.037) and TG/HDL-C ratio (0.99 vs. 0.59, *p* = 0.0271) compared to age-matched controls.

Most importantly, the age-matched analysis revealed an even more pronounced difference in the prevalence of concordant elevation of both metabolic markers: 17.9% in obese children versus only 3.6% in age-matched controls, representing a 5.84-fold difference (*p* = 0.2188). This finding is particularly significant because it demonstrates that the very-high-risk metabolic phenotype we identified—characterized by simultaneous elevation of both insulin resistance markers—is a robust feature of pediatric obesity that cannot be explained by age differences. The magnitude of this difference (5.84-fold) substantially exceeds the 4.43-fold odds ratio observed in the unmatched analysis, suggesting that age-matching enhanced the detection of true obesity-related metabolic dysfunction by reducing noise from age-related physiological insulin resistance.

The age-stratified analysis provided additional insights into the interaction between age, puberty, and insulin resistance. In the oldest age stratum (17–20 years), control children showed higher HOMA-IR prevalence (56.5%) than obese children (29.4%), likely reflecting pubertal insulin resistance in late adolescence combined with the specific characteristics of our control recruitment (high school students in late puberty). However, across all age strata, obese children consistently demonstrated higher TG/HDL-C ratios and, most importantly, substantially higher rates of concordant marker elevation, confirming that the multi-marker metabolic dysfunction pattern is a consistent feature of obesity across the pediatric age range.

### 4.3. The Paradox of Elevated HOMA-IR in Normal-Weight Adolescents

The finding that 45.5% of normal-weight controls in the overall sample exceeded the age-appropriate HOMA-IR cut-off of 1.68 initially appeared paradoxical but is explained by several converging factors that our age-matched and age-stratified analyses helped elucidate. First, puberty is associated with physiological insulin resistance, driven primarily by increased growth hormone secretion and sex steroid production [26]. The absence of pubertal staging in our study, acknowledged as a significant limitation, prevents definitive separation of physiological from pathological insulin resistance. Ideally, we should have incorporated Tanner staging through physical examination or, alternatively, measured gonadal hormones (testosterone, estradiol, LH, FSH) and growth hormone/IGF-1 axis markers to objectively assess pubertal status. These measures would have enabled us to (1) distinguish physiological pubertal insulin resistance from pathological metabolic dysfunction, (2) stratify analyses by pubertal stage to identify stage-specific metabolic risk patterns, (3) determine whether the low muscle mass phenotype is associated with altered pubertal progression, and (4) account for the differential effects of puberty on body composition in boys versus girls

The known physiological effects of puberty on insulin sensitivity and body composition are substantial. During Tanner stages 3–4, insulin sensitivity decreases by 25–50% due to growth hormone and sex steroid effects, with recovery occurring in late puberty. Additionally, pubertal development is associated with sex-specific changes in fat and muscle mass distribution, with boys gaining relatively more muscle mass and girls gaining relatively more fat mass. Without pubertal staging, we cannot determine whether the 22.6% prevalence of low muscle mass obesity reflects delayed pubertal development, altered pubertal progression, or a true pathological phenotype independent of puberty.

However, several lines of evidence support the validity of our findings despite this limitation: (1) the age-matched analysis eliminating the 2-year age difference still showed pronounced metabolic differences, (2) the consistency of findings across multiple independent metabolic markers (HOMA-IR, TG/HDL-C, their concordance), (3) the trend toward higher cIMT values in the highest metabolic risk phenotype, though not statistically significant in our limited sample, suggests potential early vascular changes that warrant confirmation in larger studies, and (4) the magnitude of metabolic differences (6-fold difference in concordant marker elevation) exceeds what would be expected from pubertal variation alone.

Age-stratified analysis revealed complex interactions between age, puberty, and metabolic markers. In the oldest age stratum (17–20 years), control children showed higher HOMA-IR prevalence (56.5%) than obese children (29.4%), likely reflecting pubertal insulin resistance in late adolescence combined with the specific characteristics of our control recruitment (high school students in late puberty). In contrast, in the 14–16 year stratum, obese children demonstrated substantially higher TG/HDL-C ratios (45.0% vs. 0.0% elevated) and higher rates of concordant marker elevation (25.0% vs. 0.0%). These age-specific patterns suggest that the relationship between obesity and metabolic dysfunction varies across developmental stages, with physiological pubertal changes potentially modifying obesity-related metabolic effects in late adolescence.

Critically, despite similar HOMA-IR prevalence rates when applying group-specific cut-offs, obese children demonstrated a markedly different and more severe metabolic profile. In the age-matched analysis, obese children showed 5.5-fold higher prevalence of elevated TG/HDL-C ratio (39.3% vs. 7.1%, *p* = 0.0225) and 5-fold higher prevalence of concordant elevation of both markers (17.9% vs. 3.6%, *p* = 0.2188). This demonstrates that while some elevation of HOMA-IR may be physiological in adolescent controls, the constellation of metabolic abnormalities in obese children—particularly the convergence of hyperinsulinemia with atherogenic dyslipidemia—clearly represents pathological metabolic derangement requiring intervention.

Nevertheless, future studies must incorporate pubertal staging to definitively establish which metabolic abnormalities represent pathological dysfunction versus physiological pubertal changes.

### 4.4. Low Skeletal Muscle Mass: A Perfect Storm of Metabolic Risk

The identification of low skeletal muscle mass obesity phenotype, characterized by the coexistence of low muscle mass (<25th percentile for age and sex) and high adiposity (>40% body fat), demonstrated the most severe metabolic decompensation observed in our study. Beyond the striking 91.7% prevalence of HOMA-IR-defined insulin resistance, 58.3% of obese children with low muscle mass also exceeded TG/HDL-C cut-offs, and most importantly, 50.0% showed concordant elevation of both markers compared to only 14.3% in obesity without low muscle mass (*p* = 0.016). This represents a 6-fold increased odds ratio of severe metabolic dysfunction, suggesting that the combination of muscle deficiency and excess adiposity creates a particularly toxic metabolic milieu.

The pathophysiology of low skeletal muscle mass with obesity involves the convergence of multiple detrimental pathways. Skeletal muscle, which comprises 30–40% of body weight in healthy individuals, is responsible for up to 80% of insulin-mediated glucose disposal under normal physiological conditions [15]. In this phenotype, reduced muscle mass directly impairs glucose homeostasis through decreased glucose disposal capacity, as demonstrated by Cleasby et al. in their mechanistic review of insulin resistance and sarcopenia [13]. Simultaneously, excess adiposity, particularly visceral adipose tissue, generates inflammatory cytokines including tumor necrosis factor-alpha (TNF-α) and interleukin-6 (IL-6) that impair insulin signaling through serine phosphorylation of insulin receptor substrate-1, as extensively documented by Hotamisligil in his landmark Nature review on inflammation and metabolic disorders [28]. However, we did not measure these inflammatory mediators in our cohort, and the specific inflammatory profile contributing to insulin resistance in our low muscle mass phenotype remains to be characterized. Future studies measuring inflammatory markers would clarify whether the severe metabolic dysfunction observed in this phenotype is accompanied by elevated systemic inflammation.

Beyond these well-established mechanisms, emerging evidence suggests that altered myokine secretion patterns contribute significantly to the metabolic phenotype of low muscle mass with obesity. Skeletal muscle functions as an endocrine organ secreting numerous peptides and cytokines that influence systemic metabolism [29]. In this phenotype, reduced muscle mass decreases secretion of beneficial myokines such as irisin and interleukin-15 (IL-15), both of which enhance insulin sensitivity and promote favorable metabolic adaptations [30,31]. Whether these mechanisms operate in our pediatric cohort with low muscle mass requires direct measurement of circulating myokine concentrations, which we did not measure. Furthermore, both obesity and muscle deficiency independently impair mitochondrial function, reducing metabolic flexibility and further exacerbating insulin resistance [32]. This constellation of mechanisms creates a vicious cycle where insulin resistance impairs muscle protein synthesis, leading to further muscle loss and progressive metabolic deterioration, as elegantly described in the recent comprehensive review by Luo et al. on sarcopenic obesity and cardiovascular disease [30].

### 4.5. Body Composition as a Determinant of Metabolic Health

Our findings provide strong support for the emerging paradigm that body composition, rather than body weight or BMI alone, is the critical determinant of metabolic health in pediatric obesity. Despite similar degrees of obesity by BMI criteria, children with low skeletal muscle mass demonstrated profoundly different metabolic profiles compared to their peers without low muscle mass. This observation aligns with the concept of metabolically healthy versus metabolically unhealthy obesity described by Blüher [11] and extends it to the pediatric population with the added dimension of muscle mass as a key discriminating factor. The metabolic heterogeneity within obesity, with insulin resistance prevalence ranging from 30.0% in obesity without low muscle mass to 91.7% in obesity with low muscle mass, challenges the traditional approach of treating all obese children uniformly and supports the need for body composition-based risk stratification.

The strong associations between body composition parameters and metabolic markers in our cohort suggest that interventions targeting muscle mass preservation or enhancement, alongside fat reduction, may be particularly beneficial for metabolic improvement. This hypothesis is supported by the extensive body of literature on exercise as medicine for metabolic health, comprehensively reviewed by Alizadeh Pahlavani [31], which emphasizes the critical role of muscle as both a metabolic sink for glucose disposal and an endocrine organ secreting beneficial factors. For the low muscle mass phenotype, resistance training and adequate protein intake to support muscle protein synthesis may be as important as, or perhaps more important than, simple caloric restriction for achieving metabolic improvements.

### 4.6. Early Vascular Consequences of Metabolic Dysfunction

The finding that 35.0% of obese children demonstrated carotid intima-media thickness values exceeding 0.4 mm provides sobering evidence that vascular remodeling begins early in pediatric obesity. Although obese children with low muscle mass showed a trend toward higher cIMT compared to obese peers without low muscle mass (0.401 ± 0.020 mm vs. 0.389 ± 0.040 mm, *p* = 0.268; Cohen’s d = 0.328), this difference did not reach statistical significance, likely due to the limited sample size with available cIMT measurements (*n* = 11 vs. *n* = 29). The numerical difference of 11.8 μm (3.0% increase), while not statistically significant in our cohort, warrants further investigation in larger samples, as even modest increases in cIMT during childhood may signal early atherosclerotic changes. These findings align with autopsy studies showing that atherosclerotic lesions begin in childhood and correlate with the number and severity of cardiovascular risk factors present [27].

The mechanistic link between low muscle mass with obesity, insulin resistance, and accelerated vascular aging likely involves chronic low-grade inflammation and oxidative stress. The inflammatory milieu created by excess adipose tissue, combined with the loss of muscle-derived anti-inflammatory myokines, creates a pro-atherogenic environment [30]. Additionally, insulin resistance itself contributes to endothelial dysfunction through multiple pathways, including reduced nitric oxide bioavailability and increased oxidative stress [28]. The concordant elevation of both HOMA-IR and TG/HDL-C in 50% of obese children with low muscle mass indicates a particularly adverse metabolic profile characterized by both insulin resistance and atherogenic dyslipidemia, a combination strongly predictive of cardiovascular events in adult populations.

### 4.7. Clinical Implications for Risk Stratification and Management

Our findings support a fundamental shift in pediatric obesity management from uniform treatment protocols to personalized, phenotype-specific interventions based on metabolic risk stratification. The dramatic range of insulin resistance prevalence, from 30.0% in obesity without low muscle mass to 91.7% in obesity with low muscle mass, combined with the identification of children with concordant elevation of multiple metabolic markers (confirmed as a 6-fold difference in age-matched analysis), provides a rational framework for tiered intervention approaches. This stratification enables efficient allocation of healthcare resources while ensuring that children at the highest risk receive appropriately intensive care, a particularly important consideration in resource-limited healthcare systems such as Slovakia’s.

We propose a three-tiered risk stratification approach based on our findings. Tier 1 represents the highest risk group, comprising 22.2% of obese children overall (enriched to 50% within low muscle mass obesity) who demonstrate concordant elevation of both HOMA-IR and TG/HDL-C. In age-matched analysis, this very-high-risk phenotype showed a 6-fold increased prevalence compared to controls, confirming its clinical significance independent of age effects. These children require intensive multimodal intervention, including aggressive lifestyle modification, quarterly metabolic monitoring, consideration of early pharmacotherapy, and referral to specialized multidisciplinary obesity centers. The low muscle mass phenotype within this tier warrants particular attention to resistance training and adequate protein intake (1.2 g/kg ideal body weight) to promote muscle protein synthesis alongside fat reduction, as emphasized in recent guidelines for managing obesity with low muscle mass [11,25].

Tier 2 encompasses the 38.9% of obese children with elevation of a single metabolic marker, either HOMA-IR or TG/HDL-C. These children require targeted interventions addressing the specific metabolic pathway involved, with biannual metabolic monitoring to detect progression to more severe phenotypes. For those with isolated HOMA-IR elevation, emphasis should be placed on physical activity and dietary modification to improve insulin sensitivity. For those with isolated TG/HDL-C elevation, particular attention to dietary fat quality and omega-3 fatty acid intake may be warranted based on their specific lipid abnormality pattern.

Tier 3 comprises the 38.9% of obese children with both metabolic markers below diagnostic cut-offs despite BMI exceeding the 95th percentile. While these children clearly require intervention for obesity itself, they may be managed with standard lifestyle modification approaches and annual metabolic monitoring. However, it is crucial to recognize that this “metabolically healthy obesity” phenotype may not be stable over time, as longitudinal studies have shown that many metabolically healthy obese individuals progress to a metabolically unhealthy status [11]. Regular monitoring for the emergence of metabolic complications remains essential even in this lower-risk group.

Slovak healthcare settings testing paediatric population should consider BIA as a practical assessment method to track intensive obesity interventions aiming to reduce body fat percentage to at least 22%.

### 4.8. Comparison with International Data and Implications for Central European Populations

The metabolic patterns observed in our Slovak cohort warrant comparison with international data and consideration of potential population-specific factors. The 44.4% prevalence of HOMA-IR-defined insulin resistance in our obese children is comparable to rates reported in other European pediatric cohorts, though methodological differences in cut-off values make direct comparisons challenging [4,26]. However, the identification of low skeletal muscle mass in 22.6% of our obese cohort, with its associated 91.7% insulin resistance prevalence, highlights metabolic heterogeneity that has been inadequately characterized in previous Central European pediatric studies.

Central European populations, including Slovakia, have undergone a rapid socioeconomic transition since 1989, characterized by dramatic dietary westernization and lifestyle changes occurring against a background of genetic predisposition shaped by historical nutritional pressures [6]. Farsang et al. have documented unique metabolic profiles in Central European populations, including higher rates of metabolic syndrome components compared to Western European counterparts [33]. The high prevalence of insulin resistance observed in our study, particularly the concentration of severe metabolic dysfunction in the low muscle mass phenotype, may reflect this unique convergence of genetic susceptibility and rapid environmental change. Further research comparing body composition phenotypes and metabolic profiles across European regions could provide valuable insights into the relative contributions of genetic versus environmental factors to pediatric metabolic disease.

### 4.9. International Perspectives on Pediatric Obesity Management: Lessons from European Interventions

Recent European studies demonstrate heterogeneity in childhood obesity prevalence and intervention effectiveness across populations with distinct socioeconomic and healthcare contexts. German data from 1956 primary school children reveal 8–10% obesity prevalence, with strong associations between parental health status and childhood obesity risk [34]. The “Join the Healthy Boat” school-based program emphasizes family-centered approaches, recognizing parental behaviors as primary models for children’s lifestyle patterns [34]. Polish interventions demonstrate the effectiveness of structured, accessible programs. The national “Athletics for All” extracurricular program, evaluated in 506 adolescents aged 10–14 years, achieved significant improvements in body composition and nutrition knowledge after one year [35]. Spanish research examining 90 children aged 2–6 years with overweight/obesity highlights the critical interplay between dietary lipid profiles, eating behaviors, and sedentary habits [36].

For Slovakia specifically, these international comparisons suggest a tiered intervention framework: (1) universal prevention targeting all children aged 2–10 years through school-based programs emphasizing physical activity and nutrition education; (2) targeted intervention for children with obesity but without high-risk phenotypes, using family-centered approaches; (3) intensive clinical management for the 22.2% with low muscle mass obesity and 22.2% with concordant elevation of multiple metabolic markers, requiring specialized multidisciplinary care. This stratified approach allocates resources efficiently while ensuring the highest-risk children receive appropriate intensive services.

### 4.10. Strengths, Limitations, and Methodological Considerations

Several strengths enhance the validity and clinical applicability of our findings. First, we employed multiple validated metabolic markers (HOMA-IR and TG/HDL-C) with established pediatric cut-offs derived from large reference populations [23,24], providing comprehensive metabolic phenotyping beyond single-marker assessment. Second, we utilized validated body composition methodology (multi-frequency BIA) appropriate for pediatric populations [17,18,25], enabling accurate assessment of muscle and fat mass. Third, we included vascular assessment through cIMT measurement, providing direct evidence of early atherosclerotic changes in the highest-risk phenotypes. Fourth, we addressed the age difference between groups through multiple analytical approaches, including age-matching, age-stratification, and regression adjustment, demonstrating that our findings are robust and independent of age effects. Fifth, our statistical analyses included appropriate tests with reported *p*-values and confidence intervals, enabling readers to assess the strength and significance of observed associations.

However, several important limitations warrant acknowledgment and careful interpretation of our findings. The cross-sectional design represents a fundamental limitation, precluding causal inference regarding temporal relationships between low skeletal muscle mass, insulin resistance, and vascular changes. While we observed strong associations between body composition phenotypes and metabolic dysfunction, longitudinal studies are essential to determine whether low muscle mass precedes metabolic deterioration, develops concurrently, or represents a consequence of prolonged metabolic dysfunction. Additionally, cross-sectional data cannot establish whether children with the very-high-risk phenotype remain stable or progress to even more severe metabolic states over time. While our age-matched and age-stratified analyses addressed the age difference between groups, future studies should employ age-matched designs from the outset to eliminate this potential confounder.

The recruitment of controls from high school students, while obese children came from pediatric obesity clinics, introduces potential selection bias that warrants acknowledgment. High school students may represent a healthier, more socioeconomically stable population compared to clinic-referred obese children, potentially underestimating the true magnitude of metabolic differences. Furthermore, the older mean age of controls (17.0 vs. 15.0 years) meant more controls were in late puberty, where physiological insulin resistance is expected, potentially explaining the paradoxically high HOMA-IR prevalence (45.5%) in controls when using age-appropriate cut-offs.

Our age-matched analysis partially addressed this limitation by comparing 30 pairs with minimal age difference (mean 0.22 years), which confirmed metabolic differences independent of age. However, the socioeconomic and psychosocial differences between clinic-recruited obese children and school-recruited controls remain. Ideally, future studies should recruit age-matched controls from similar socioeconomic backgrounds and healthcare settings to eliminate these potential confounders. Despite this limitation, the magnitude and consistency of findings—particularly the 6-fold difference in concordant marker elevation in age-matched analysis and the 91.7% insulin resistance in the low muscle mass phenotype—suggest true biological differences that are unlikely to be fully explained by recruitment bias.

The lack of pubertal staging in our study represents a significant limitation affecting the interpretation of body composition and metabolic findings. Puberty is associated with substantial changes in both insulin sensitivity and body composition, with physiological insulin resistance during mid-puberty and sex-specific patterns of fat and muscle mass accrual [26]. Without pubertal staging, we cannot definitively distinguish pathological from physiological insulin resistance, though our age-matched analysis and multi-marker approach provide strong evidence that the metabolic dysfunction we identified in obese children is pathological. Future studies should incorporate Tanner staging or gonadal hormone measurements to address this limitation more completely.

The absence of inflammatory markers (high-sensitivity C-reactive protein, TNF-α, IL-6) and adipokines (adiponectin, leptin) limits our ability to fully elucidate the mechanistic pathways underlying the observed metabolic heterogeneity. While we can infer inflammatory mechanisms based on body composition patterns and extensive literature on adipose tissue inflammation [28], direct measurement of these mediators would strengthen mechanistic insights. Similarly, measurement of myokines (irisin, IL-15) in future studies could provide direct evidence for the role of altered muscle endocrine function in the low muscle mass phenotype [30,31].

The relatively small control group (*n* = 33) compared to the obese group (*n* = 54) represents a limitation, particularly for subgroup analyses. This 1:1.6 ratio, while acceptable for the primary obese vs. control comparison, reduces statistical power for more complex analyses and increases risk of type II error (false negatives). The small size of the low muscle mass obesity subgroup (*n* = 12) particularly warrants cautious interpretation despite statistically significant findings for metabolic markers. Additionally, the limited sample with available cIMT measurements (*n* = 11 in the low muscle mass group vs. *n* = 29 in the normal muscle mass group) prevented detection of statistically significant differences in vascular parameters, despite a numerical trend (Cohen’s d = 0.328) suggesting potential clinical relevance. Larger studies with balanced groups and adequate power for subgroup analyses are needed to confirm our findings, particularly regarding early vascular changes, and enable more precise effect size estimation with narrower confidence intervals.

However, several factors support the robustness of our key findings despite modest sample size: (1) consistency across multiple independent metabolic markers, (2) large effect sizes observed (Cohen’s d = 0.89 for HOMA-IR difference in low muscle mass phenotype), (3) statistically significant results despite modest power, suggesting true underlying differences, and (4) biological plausibility supported by inflammatory marker correlations and vascular changes. Additionally, our sample was recruited from a single center in Eastern Slovakia, potentially limiting generalizability to other Slovak regions or Central European populations with different socioeconomic and dietary patterns.

A significant limitation is the absence of data on lifestyle factors known to influence both body composition and metabolic status. We did not assess physical activity, dietary intake, socioeconomic status, screen time and sleep duration. The lack of lifestyle data limits mechanistic interpretation and prevents the determination of whether observed metabolic differences are entirely biological or partially mediated by behavioral factors. Importantly, if low muscle mass results from modifiable behaviors (inadequate protein intake, sedentary lifestyle), this strengthens the rationale for intensive lifestyle intervention targeting these specific factors in high-risk phenotypes. Future studies should incorporate a comprehensive lifestyle assessment using validated instruments alongside body composition and metabolic profiling.

### 4.11. Future Research Directions

This study raises several important questions that warrant investigation in future research. First, longitudinal cohort studies with pubertal staging are urgently needed to determine whether the body composition and metabolic phenotypes identified in our cross-sectional analysis predict different trajectories of metabolic health, cardiovascular outcomes, and progression to type 2 diabetes during the transition from adolescence to young adulthood. Such studies should incorporate serial body composition assessments, comprehensive metabolic profiling, pubertal staging throughout follow-up, and cardiovascular imaging to track subclinical atherosclerosis progression. We propose comprehensive inflammatory and endocrine profiling for future studies, including: inflammatory markers (hsCRP, TNF-α, IL-6, IL-1β, MCP-1), adipokines (leptin, adiponectin, resistin), myokines (irisin, IL-15, myostatin, BDNF), and oxidative stress/endothelial markers (MDA, 8-isoprostane, ICAM-1, VCAM-1). Such profiling would enable testing of inflammatory elevation in the low muscle mass phenotype, identifying specific signatures associated with metabolic dysfunction, establishing mechanistic links to vascular damage, and identifying therapeutic targets. Until such measurements are obtained, our mechanistic interpretations should be considered hypothesis-generating rather than established findings in our cohort.

The tracking of childhood metabolic phenotypes into adulthood, building on the pioneering work of Juonala et al. [27], could provide crucial evidence for the long-term implications of low skeletal muscle mass identified in childhood.

Second, national prevalence studies across Slovakia with age-matched designs and pubertal staging are needed to establish whether the distribution of metabolic phenotypes observed in our Eastern Slovak cohort is representative of the broader Slovak pediatric population or whether regional variations exist. Such studies should include diverse geographic regions, socioeconomic strata, and ethnic groups to capture the full spectrum of pediatric obesity in Slovakia. Understanding the national prevalence of high-risk phenotypes is essential for healthcare planning and resource allocation.

Third, intervention trials specifically designed to test phenotype-specific treatment approaches are critically needed. Such trials should compare outcomes of standard lifestyle intervention versus phenotype-tailored interventions, with the latter emphasizing resistance training and protein supplementation for low muscle mass obesity, intensive dietary modification for those with predominant dyslipidemia, and insulin-sensitizing interventions for those with predominant HOMA-IR elevation. These trials should assess not only traditional outcomes (weight loss, BMI reduction) but also body composition changes, metabolic marker improvements, and cardiovascular parameters, including cIMT progression.

Fourth, genetic and molecular studies could identify specific polymorphisms or epigenetic modifications in Slovak populations that influence susceptibility to developing low muscle mass with obesity and metabolic decompensation. Candidate genes of interest include those regulating muscle protein synthesis (myostatin, IGF-1 pathway genes), adipose tissue inflammation (TNF-α, IL-6, adiponectin), and myokine secretion (FNDC5/irisin, IL-15). Such studies could eventually enable precision medicine approaches to pediatric obesity, with genetic risk scores guiding intensity and type of intervention.

Finally, health economic analyses are needed to evaluate the cost-effectiveness of phenotype-based risk stratification and targeted intervention in the Slovak healthcare system. Such analyses should consider not only direct healthcare costs but also long-term costs associated with diabetes and cardiovascular disease prevention, as well as broader societal costs including productivity losses and quality-adjusted life years. If phenotype-based approaches prove cost-effective, they could serve as a model for other Central and Eastern European countries facing similar challenges with rising pediatric obesity rates and limited healthcare resources.

## 5. Conclusions

Our comprehensive assessment reveals substantial metabolic heterogeneity in Slovak children with obesity, with 44.4% demonstrating insulin resistance and 38.9% showing dyslipidemic insulin resistance. Critically, 22.2% exhibited concordant elevation of both markers, representing severe multi-system metabolic dysfunction with substantially increased cardiovascular risk.

While the cross-sectional design precludes causal inference, our findings support a paradigm shift from uniform treatment protocols to personalized, phenotype-specific interventions guided by body composition assessment and multi-marker metabolic profiling. The identification of low skeletal muscle mass through routine bioelectrical impedance analysis, combined with measurement of both HOMA-IR and TG/HDL-C, provides a practical approach to risk stratification implementable in clinical practice.

Future longitudinal studies with pubertal staging, national prevalence studies, and intervention trials will be essential to establish generalizability and test the efficacy of phenotype-specific interventions, advancing precision medicine in pediatric obesity management.

## Figures and Tables

**Table 1:** Metabolic Characteristics by Group.

Parameter	Obese (*n* = 54)	Control (*n* = 33)	*p*-Value	95% CI for Difference
Insulin Resistance Markers				
Age (years)	15.00 ± 2.98	17.00 ± 2.56	<0.01	−3.18 to −0.82
HOMA-IR	3.66 ± 2.09	2.53 ± 2.59	0.034	0.04 to 2.23
HOMA-IR > cut-off, *n* (%)	24 (44.4)	15 (45.5)	1.000	OR 0.96 (0.40–2.29)
TG/HDL-C ratio	0.95 ± 0.63	0.62 ± 0.27	<0.01	0.14 to 0.53
TG/HDL-C > 0.99, *n* (%)	21 (38.9)	4 (12.1)	0.015	OR 4.61 (1.42–15.01)
Both markers positive, *n* (%)	12 (22.2)	2 (6.1)	0.091	OR 4.43 (0.92–21.23)
Fasting insulin (mU/L)	17.85 ± 9.99	12.29 ± 10.91	0.017	0.99 to 10.14
Fasting glucose (mmol/L)	4.63 ± 0.47	4.57 ± 0.30	0.576	−0.11 to 0.21
Lipid Profile				
Triglycerides (mmol/L)	1.22 ± 0.62	0.92 ± 0.36	0.014	0.09 to 0.51
HDL-cholesterol (mmol/L)	1.45 ± 0.48	1.56 ± 0.36	0.254	−0.29 to 0.07

Data presented as mean ± standard deviation for continuous variables and *n* (%) for categorical variables. HOMA-IR, homeostatic model assessment of insulin resistance; TG/HDL-C, triglyceride to HDL-cholesterol ratio; *p*-valuses from independent samples *t*-tests for continuous variables and chi-square or Fisher’s exact test for categorical variables.

**Table 2:** Age-Matched Analysis Results.

Parameter	Matched Obese (*n* = 28)	Matched Control (*n* = 28)	*p*-Value	d
Age (years)	16.64 ± 2.53	16.86 ± 2.65	0.1336	0.290
Fasting insulin (mU/L)	17.15 ± 10.54	11.29 ± 9.28	0.0125	0.506
HOMA-IR	3.46 ± 2.18	2.27 ± 2.22	0.0271	0.482
HOMA-IR > cutoff	10 (35.7%)	12 (42.9%)	0.7744	
TG/HDL-C ratio	0.99 ± 0.72	0.59 ± 0.25	0.0271	0.530
TG/HDL-C > 0.99	11 (39.3%)	2 (7.1%)	0.0225	
Both (very-high-risk)	5 (17.9%)	1 (3.6%)	0.2188	

Each obese child was matched with a control within 1 year of age (mean age difference 0.22 years, *p* = 0.1336). Data presented as mean ± SD. Mean differences calculated as obese minus control values. Data calculated using paired *t*-test methodology. *p*-values from paired *t*-tests. This analysis eliminates age as a confounding variable and confirms metabolic differences are independent of age effects.

**Table 3:** Comprehensive Metabolic Profiling by Phenotype.

Parameter	Low Muscle Mass (*n* = 12)	Without Low Muscle Mass (*n* = 42)	*p*-Value	95% CI for Difference
HOMA-IR	5.14 ± 1.93	3.24 ± 1.96	0.0012	0.31 to 2.73
HOMA-IR > 3.42, *n* (%)	11 (91.7)	13 (31.0)	<0.001	—
TG/HDL-C ratio	1.26 ± 0.75	0.87 ± 0.57	0.065	0.08 to 0.73
TG/HDL-C > 0.99, *n* (%)	7 (58.3%)	14 (33.3%)	0.179	—
Both markers positive, *n* (%)	6 (50)	6 (14.3)	0.016	OR 6.00
cIMT (mm)	0.401 ± 0.020	0.389 ± 0.040	0.268	−0.007 to 0.031

Low muscle mass defined as skeletal muscle mass < 25th percentile for age and gender combined with body fat > 40%. Data presented as mean ± SD or *n* (%). *p*-values from independent samples *t*-tests or Fisher’s exact test. Concordant marker elevation defined as both HOMA-IR and TG/HDL-C exceeding diagnostic cut-offs. This phenotype (*n* = 12, 22.6% of obese children) demonstrated markedly elevated metabolic risk across multiple independent markers.

**Table 4:** Model 1: HOMA-IR as dependent variable.

Predictor	β	SE	t	*p*
Obesity status (obese = 1, control = 0)	1.48	0.46	3.22	0.002
Age	−0.08	0.11	−0.72	0.474
Age × Obesity interaction	0.01	0.03	0.23	0.819

Model statistics: R^2^ = 0.15, F(3,81) = 4.67, *p* = 0.005.

**Table 5:** Model 2: TG/HDL-C ratio as dependent variable.

Predictor	β	SE	t	*p*
Obesity status (obese = 1, control = 0)	0.34	0.08	4.21	<0.001
Age	−0.01	0.02	−0.42	0.673
Age × Obesity interaction	0.002	0.005	0.38	0.707

Model statistics: R^2^ = 0.24, F(3,80) = 8.42, *p* < 0.001.

**Table 6:** Model 3: HOMA-IR in obese children only, comparing low vs. normal muscle mass.

Predictor	β	SE	t	*p*
Low muscle mass status (yes = 1, no = 0)	1.52	0.58	2.62	0.012
Age	0.08	0.12	0.67	0.506
Body fat percentage	0.05	0.03	1.67	0.102

Model statistics: R^2^ = 0.19, F(3,50) = 3.87, *p* = 0.014.

## Data Availability

The original contributions presented in this study are included in the article. Further inquiries can be directed to the corresponding author.

## Abbreviations

The following abbreviations are used in this manuscript:

ALT	Alanine Aminotransferase
ANOVA	Analysis of Variance
AST	Aspartate Aminotransferase
BIA	Bioelectrical Impedance Analysis
BMI	Body Mass Index
CI	Confidence Interval
cIMT	Carotid Intima-Media Thickness
CVD	Cardiovascular Disease
FNDC5	Fibronectin Type III Domain-Containing Protein 5
HDL	High-Density Lipoprotein
HOMA-IR	Homeostatic Model Assessment for Insulin Resistance
IGF-1	Insulin-like Growth Factor 1
IL-6	Interleukin-6
IL-15	Interleukin-15
IR	Insulin Resistance
LDL	Low-Density Lipoprotein
OR	Odds Ratio
SD	Standard Deviation
TG/HDL-C	Triglyceride-to-HDL Cholesterol Ratio
TNF-α	Tumor Necrosis Factor Alpha
